# Demystifying the Complexity: A Case Report on the Management of Mandibular Canine with Two Roots

**DOI:** 10.7759/cureus.7072

**Published:** 2020-02-21

**Authors:** Akhilesh Bhaskar, Rega Kumar, Ruchika R Nawal, Sangeeta Talwar

**Affiliations:** 1 Conservative Dentistry & Endodontics, Maulana Azad Institute of Dental Sciences, New Delhi, IND

**Keywords:** mandibular canine, two roots, anatomical aberrations

## Abstract

Mandibular canine often presents with only one root and one root canal in most of the cases. However, approximately 15% of the mandibular canines have two root canals and even less frequently two distinct roots. This article presents a case of root canal treatment in mandibular canine with two roots and two root canals. Meticulous cleaning and shaping followed by three-dimensional filling help in the successful treatment of such anatomical aberrations. Failure to do so may lead to post-treatment disease and further complications.

## Introduction

The aim of root canal treatment is to remove vital and necrotic tissues from the root canal system and prevent reinfection by the development of an impermeable seal [1]. The complexity of the root canal system necessitates the clinicians to be aware of the internal anatomy before starting endodontic treatment [2]. The mandibular canine has Vertucci’s type I configuration in approximately 85% of the cases [3-5]. However, cases may present with two canals in a single root or even less commonly, with two roots and two root canals and inadequate knowledge of such anatomical variations is one of the most common causes of endodontic treatment failure [6-10]. A patient presenting with such root canal anatomy is unpredictable, and the clinician should always be recognizant of such variations that may affect the prognosis.

## Case presentation

**Figure 1:**
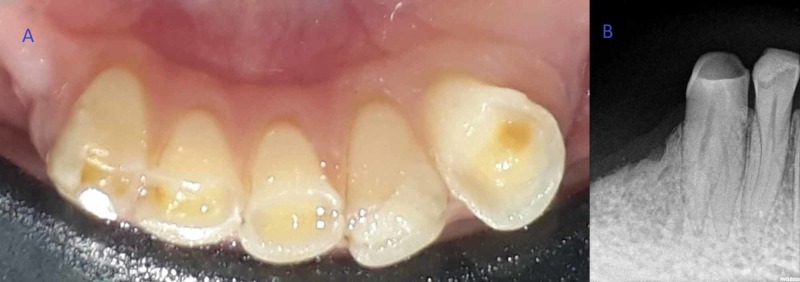
Preoperative images. (A) Preoperative clinical photograph of the mandibular arch. (B) Preoperative radiograph of mandibular canine with two roots.

A 35-year-old female patient reported to the department of conservative dentistry and endodontics with pain in the right lower front tooth region for the past month. Medical history was unremarkable. On clinical examination, there was severe attrition with dentinal exposure and microcracks in enamel in relation to tooth number 43 (Figure 1A). Oral mucosa was normal with no presence of any sinus tracts. On pulp sensibility testing (electric pulp test and cold test), tooth number 43 showed an exaggerated response. On examination of radiographs taken at multiple angulations, it was observed that root was bifurcating into two roots at the coronal third of mandibular canine (Figure 1B).

Based on history, clinical examination, and investigations, a diagnosis of symptomatic irreversible pulpitis was made for tooth number 43. Root canal treatment was advised and informed consent was taken from the patient. Local anesthesia (2% lidocaine hydrochloride with 1:80,000 epinephrine) (Lignospan Special, Septodont, Raigad, India) was administered, and a dental dam was placed. 

**Figure 2:**
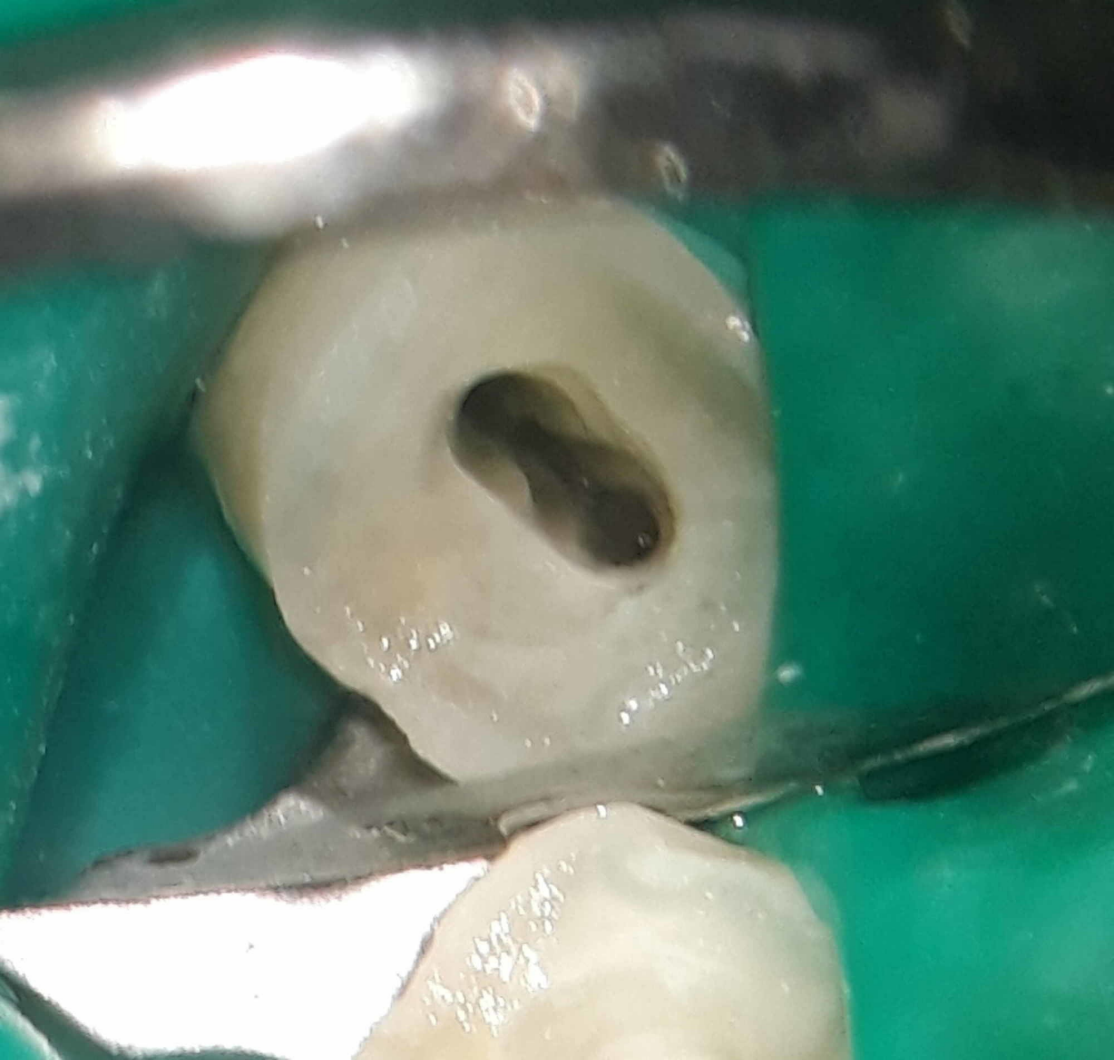
Access opening under the dental operating microscope showing two distinct canal orifices.

All procedures were performed under the dental operating microscope (OPMI PROergo, Carl Zeiss, Oberkochen, Germany) for better magnification and illumination. The access cavity was prepared, and DG-16 endodontic explorer was used to determine the bifurcation level of the root in mandibular canine. Ultrasonic tips (ProUltra Endo Tips, Dentsply Maillefer, Ballaigues, Switzerland) were then used to remove pericervical dentin obstructing the path to lingual root in mandibular canine (Figure 2).

**Figure 3:**
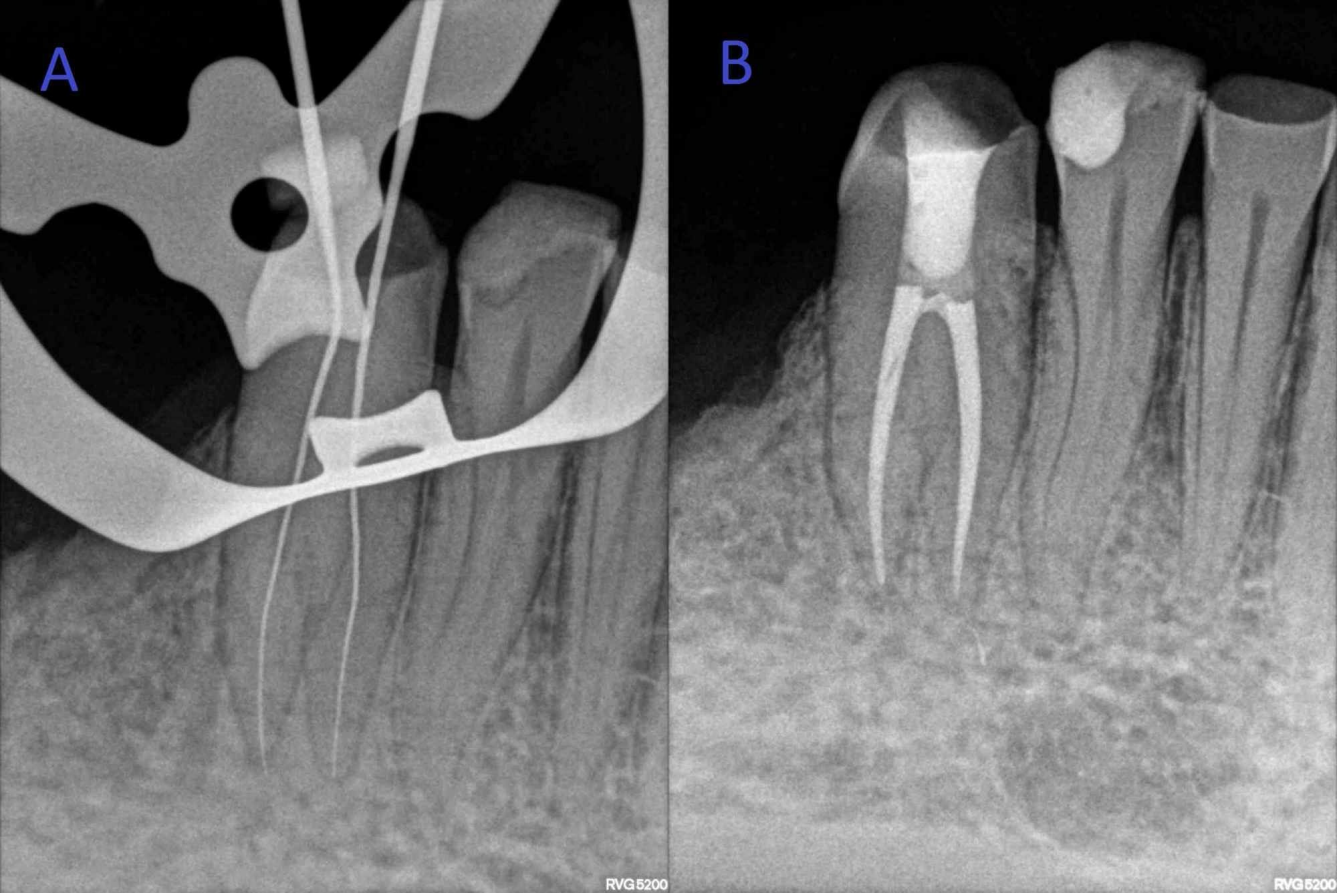
(A) Working length radiograph. (B) Postoperative radiograph.

After gaining straight-line access, the root canal was irrigated with 3% sodium hypochlorite (Parcan, Septodont, Raigad, India), and #15 size K-files (Dentsply Maillefer, Ballaigues, Switzerland) were used to determine working length using electronic apex locator (Dentaport ZX, J Morita, Kyoto, Japan) which was then reconfirmed using radiographs (Figure 3A). Biomechanical preparation was completed using rotary nickel-titanium files (ProTaper Gold, Dentsply Maillefer, Ballaigues, Switzerland) till apical file size #30 under abundant irrigation with 3% sodium hypochlorite. It was followed by irrigation with 17% EDTA to remove inorganic tissue. Master cone radiograph was taken, and obturation was done using gutta-percha cones and epoxy resin sealer (AH Plus, Dentsply, Konstanz, Germany), using the lateral cold compaction technique. Post endodontic restoration was done using composite resin (Filtek Z250 XT, 3M ESPE, St. Paul, MN) (Figure 3B).

**Figure 4:**
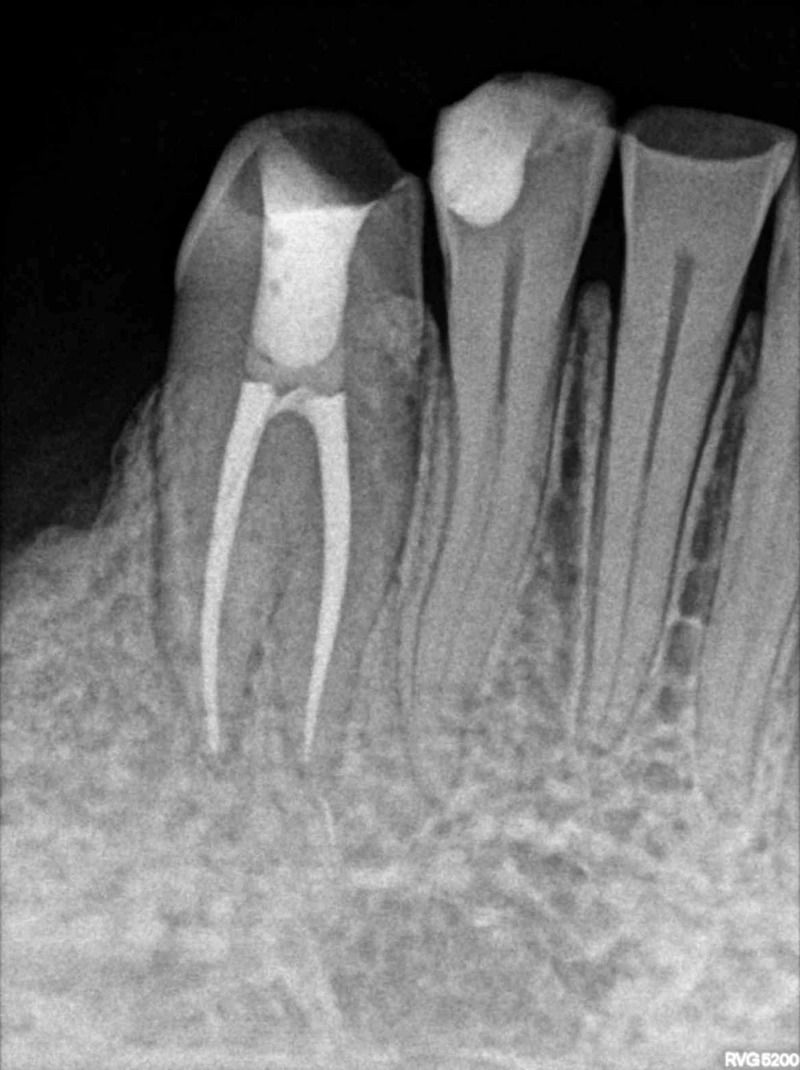
Postoperative follow-up radiograph at six months.

On follow-up after six months, the patient was asymptomatic with no signs of any periapical changes (Figure 4).

## Discussion

The complex nature of the root canal system prerequisites the clinician to scrutinize for the presence of any anatomical variation. Morphologically, mandibular canines usually have only one root and one root canal in approximately 85% of the cases [7]. However, atypical findings like mandibular canine having one root with two root canals and two roots with two root canals have been reported [6-9,11]. In fact, a rare case of mandibular canine with two roots and three root canals has been reported by Heling et al [12].

Careful inspection of diagnostic radiographs is very important during the diagnosis of such anatomical peculiarities. If some anatomical variation is suspected, angled radiographs (Clark’s technique) can be used [13,14]. Cone-beam computed tomography gives accurate detail in axial, sagittal, and coronal planes and is recommended if the diagnosis cannot be made using conventional radiographs [15]. Access opening providing straight-line access to the pulp chamber is recommended for successful endodontic treatment [7]. The anomalous presence of bifurcation at different levels mandates the clinician to perform endodontic procedures with adjunct magnifying aids. The dental operating microscope helps in the location of the exact division point and ultrasonics can then be used to gain access to lingual root without removing excess tooth structure. On the other hand, two-rooted mandibular canines may have apical foramen deviated in the buccal or lingual plane, which makes accurate working length determination difficult using radiographs alone [16]. Thus, an electronic apex locator is recommended for the precise location of apical constriction.

After gaining access to the root canal system, cleaning and shaping were done using rotary instruments in a crown down manner to minimize extrusion of debris [17]. Ultrasonic activation of irrigating solution enhances the removal of bacteria and dentin debris, which is followed by obturation to seal inaccessible anatomical zones [18].

## Conclusions

This case report emphasizes that clinicians should be aware of anatomical variations that can occur in teeth which they are treating. The presence of sudden narrowing of the root canal lumen or radiolucent groove in the lateral part of the root on radiographic examination suggests the presence of a mandibular canine with two roots and two root canals.

It is also highly recommended for the clinician to use appropriate equipment and techniques to manage such teeth with anatomical variations.We suggest to perform endodontic procedure for such teeth under the dental operating microscope and ultrasonic tips for conservative access opening and electronic apex locator for accurate working length determination.
